# Analysis of Marrubiin in *Marrubium alysson* L. Extract Using Advanced HPTLC: Chemical Profiling, Acetylcholinesterase Inhibitory Activity, and Molecular Docking

**DOI:** 10.3390/metabo14010027

**Published:** 2023-12-30

**Authors:** Nermeen A. Eltahawy, Asmaa I. Ali, Salma A. Ibrahim, Mohamed S. Nafie, Amal M. Sindi, Hanaa Alkharobi, Ahmad J. Almalki, Jihan M. Badr, Sameh S. Elhady, Reda F. A. Abdelhameed

**Affiliations:** 1 Department of Pharmacognosy, Faculty of Pharmacy, Suez Canal University, Ismailia 41522, Egypt; nermeen.azmy@pharm.suez.edu.eg (N.A.E.); reda.fouad@gu.edu.eg (R.F.A.A.); 2 Department of Pharmacognosy, Faculty of Pharmacy, Misr International University, Cairo 12585, Egypt; asmaa.ibrahim@miuegypt.edu.eg (A.I.A.); salma.ibrahim@miuegypt.edu.eg (S.A.I.); 3 Department of Chemistry, College of Sciences, University of Sharjah, Sharjah P.O. Box 27272, United Arab Emirates; 4 Chemistry Department, Faculty of Science, Suez Canal University, Ismailia 41522, Egypt; 5 Department of Oral Diagnostic Sciences, Faculty of Dentistry, King Abdulaziz University, Jeddah 21589, Saudi Arabia; amsindi@kau.edu.sa; 6 Department of Oral Biology, Faculty of Dentistry, King Abdulaziz University, Jeddah 21589, Saudi Arabia; halkharobi@kau.edu.sa; 7 Department of Pharmaceutical Chemistry, Faculty of Pharmacy, King Abdulaziz University, Jeddah 21589, Saudi Arabia; 8 Department of Natural Products, Faculty of Pharmacy, King Abdulaziz University, Jeddah 21589, Saudi Arabia; 9 Department of Pharmacognosy, Faculty of Pharmacy, Galala University, New Galala 43713, Egypt

**Keywords:** *M. alysson* L., GC/MS, HPTLC, marrubiin, acetylcholinesterase inhibitory activity, healthcare, molecular docking

## Abstract

The main purpose of this work is to investigate the phytochemical composition of *Marrubium alysson* L. non-polar fraction. GC/MS analysis was used to evaluate the plant extract’s saponifiable and unsaponifiable matter. Although *M. alysson* L. lipoidal matter saponification produced 30.3% of fatty acid methyl esters and 69.7% of unsaponifiable matter. Phytol was the most dominant substance in the unsaponifiable materials. Notably, marrubiin which is one of the most prominent metabolites of *Marrubium alysson* L. was not detected through our adopted GC/MS technique. Thus, further characterization was proceeded through simple and rapid HPTLC analysis which successfully managed to identify marrubiin. Based on the regression equation, the concentration of marrubiin in *M. alysson* L. extract was 14.09 mg/g of dry extract. Concerning acetylcholinesterase inhibitory activity, both the crude *M. alysson* L. total methanolic extract and the non-polar fraction displayed reasonable inhibitory activity against acetylcholinesterase (AChE), whereas the pure compound marrubiin was considered to be the most effective and potent AChE inhibitor, with an IC_50_ value of 52.66 (µM). According to the molecular docking studies, potential sites of interaction between the pure chemical marrubiin and AChE were examined. The results show that Tyr124 on AChE residue was critical to the activity of the aforementioned drug. Based on the depicted marrubin AChE inhibition activity and reported safety profile, this chemical metabolite is considered as a promising lead compound for further pre-clinical investigation as well as drug development and optimization.

## 1. Introduction

*Marrubium alysson* L. (*M. alysson* L.), sometimes referred to as hashisha rabiah or white horehound, is widely distributed in Egypt. Moreover, *M. alysson* L. grows in the Sinai desert and is also found in the Mediterranean coastal zone and North Africa [1]. *Marrubium* also grows in European, Asian, and North African temperate zones, and some other *Marrubium* species grow in North America and South America [2]. The family Lamiaceae, sometimes referred to as the Labiatae or mint family, to which *M. alysson* L. belongs, is one of the most essential herbal families and contains numerous perennial plants. There are more than 240 genera and more than 7200 species in this family distributed worldwide with highly valuable biological activity [3]. Since ancient times, many plants belonging to this family have been used in the form of tinctures or infusions to treat a variety of diseases [1]. Several species within the genus *Marrubium* are traditionally used as medicinal plants and have potential therapeutic activity due to the reported antispasmodic, hypolipidemic, hypotensive, hypoglycemic, and inflammatory inhibitory effects and analgesic activity [4]. The genus *Marrubium* incorporates a wide variety of many classes of compounds, such as flavonoids, volatile oils, alkaloids, phenylpropanoids, and labdane diterpenes [5]. Several diterpenes, sterols, flavonoids, and phenolic compounds were previously isolated from *M. alysson* L. [6]. Marrubiin, the first of these diterpenoids to be isolated and the most well known, contains a ring called γ-lactone that may be opened by alkaline hydrolysis to produce marrubic acid [7]. Marrubiin is considered a compound of potential value and a key component of numerous species of the genus *Marrubium* [8]. Initially, it was isolated from *Marrubium vulgare* and numerous medicinal plants of the mint family, such as *Marrubium alysson* L. [9]. This compound is the most active diterpenoid and showed effective biological properties and a high safety profile [9]. The labdane skeleton displayed by marrubiin is a characteristic representative marker of the genus *Marrubium* [10]. Furthermore, it is characterized by a low rate of turnover, is extremely stable, and has minimal catabolism, which is a crucial property required for various potent medicinal drugs [8]. It has been reported that many pharmacological studies have shown that marrubiin has antihyperalgesic [11], antioedematogenic [12], and cardioprotective effects [9]; is a free radical scavenger, countering genotoxic activity [13,14]; and shows gastroprotective [15], analgesic [16], vasorelaxant [17], and immunomodulating activity [18]. Upon administration to rabbit jejunum, an antispasmodic potential of marrubiin was noticed [19]. Anticoagulant, calcium antagonist, and antithrombotic activities have also been reported [14,20]. Moreover, a general inhibitory impact of marrubiin on several phlogistic agents was demonstrated [21]. This labdane lactone also showed increasing LDL cholesterol and insulin secretion [2]. Alzheimer’s disease (AD) is a neurological condition that impairs memory and other cognitive functions. Alzheimer’s is one of the most serious risks facing the elderly population [22]. The primary purpose of the serine hydrolase enzyme acetylcholinesterase is to hydrolyze acetylcholine in order to control the transmission of cholinergic signals. Acetylcholine, a neurotransmitter, is hydrolyzed by the enzyme into two inactive molecules: acetic acid and choline [23,24]. Memory loss and cognitive dysfunctions are partially reversed by inhibiting acetylcholinesterase in the brain through increasing acetylcholine concentration [25]. Galantamine, donezepil, tacrine, and rivastigmine are among the medications used the most to treat Alzheimer’s disease and are acetylcholinesterase activity inhibitors [26]. Controlling the amount of acetylcholine, a neurotransmitter in cholinergic synapses, is one of the most crucial therapeutic methods in the treatment of neurodegenerative diseases, and its breakdown is limited using acetylcholinesterase inhibitors [27]. Therefore, finding some potent acetylcholinesterase inhibitors may therefore be crucial for developing novel, effective therapies for neurodegenerative disorders. In a previous study, we extensively studied *M. alysson* L. methanolic extract and its polyphenolic fraction both chemically and in terms of its protective effect against mice testicles that were damaged by methotrexate through regulation of the expression of miRNA-29a and apoptosis [28]. We focus on the non-polar portion of *M. alysson* L. in the current study (n-hexane extract). This portion is investigated chemically using GC/MS analysis and the cholinesterase inhibitory potential. The same effect is studied for marrubiin as well as its quantitation using a developed HPTLC method.

## 2. Materials and Methods

### 2.1. Analysis of n-Hexane Extract Using GC-MS

#### 2.1.1. Chemicals

Solvents of analytical grade were used for extraction and fractionation. Before usage, the solvents were subjected to distillation, whereas for GC-MS analysis, HPLC-grade solvents were used.

#### 2.1.2. Extraction and Fractionation of the Plant

In April 2017, *Marrubium alysson* L. was collected from Burg El Arab (Alexandria). At Alexandria University’s Faculty of Science, the plant was identified. At Suez Canal University’s Faculty of Pharmacy’s Pharmacognosy Department, a voucher Specific was kept with the code Marr-2017. After collection of the plant, it was air dried and converted to fine powder. A total of 3 × 1 L of 70% methanol was used to extract one kilogram of plant powder. The yield of the extract was 231 g. Then, the combined methanol extract was vacuum concentrated to produce extract residue. The residue was fractionated using an open-column method with gradient elution of n-hexane, ethyl acetate, and chloroform to yield a residue of 39.42 g of the hexane fraction, 19.96 g of the ethyl acetate fraction, and 66.63 g of the chloroform fraction. Additionally, fractions of various polarity were evaporated in a vacuum and weighed. Analysis using GC-MS (gas chromatography–mass spectrometry) was performed on the resultant n-hexane fraction.

#### 2.1.3. Preparation of Unsaponifiable Matter

The saponification process was applied to around 250 mg of the n-hexane fraction by refluxing it for 24 h at 90 °C with 50 mL of 10% ethanolic potassium hydroxide and 20 mL of benzene. The resulting mixture was vacuum evaporated until it reached its third volume of concentration. The resulting residue was mixed with 100 mL of distilled water. Using a separating funnel, the aqueous solution was repeatedly partitioned against diethyl ether, and this process was continued until all of the unsaponifiable material was removed. Following a series of rinses with distilled water, the recovered diethyl ether extracts were then dried over anhydrous sodium sulphate. There were 80 mg of unsaponifiable materials in the final dry residue [6].

#### 2.1.4. Preparation of Fatty Acid Methyl Ester

The pH was determined using a litmus paper as an indicator, and the aqueous alkaline solution that was left after removing the unsaponifiable matter was acidified with concentrated HCl until it became neutral or mildly acidic. After that, the free fatty acids were repeatedly and thoroughly removed with ether using a separating funnel. Several washes with distilled water were performed on the ether extracts until the wash no longer affected the color of the litmus paper. Then, they were dried over anhydrous sodium sulphate. The total amount of free fatty acids in the final residue was 50 mg.

Fatty acid methyl ester was prepared by heating 20 mg of the final residue at 85 °C for 120 min while being refluxed with 100% methanol (50 mL) and concentrated H_2_SO_4_ (2.5 mL). After cooling, the mixture was diluted with 100 mL of distilled water and repeatedly extracted with diethyl ether using a separating funnel to extract all of the fatty acid methyl esters. The collected diethyl ether extracts were then repeatedly washed with distilled water until the wash was no longer reactive with litmus paper and then dried over anhydrous sodium sulphate [6].

#### 2.1.5. GC-MS Analysis

A split–splitless injector, a Shimadzu GCMS-QP2010 outfitted with an RTX-5 fused bonded column (30 m × 0.25 mm i.d. × 0.25 m film thickness), and a Restek GCMS-QP2010 were utilized to record the mass spectra. The operational parameters and settings for the analyses of the unsaponifiable matter and the saponifiable fraction were changed and adjusted in accordance with the previously described approach [29]. Standard alkanes (C_7_-C_40_) obtained from Sigma-Aldrich-Merck-Germany, were employed to calculate retention index (RI). The Wiley Registry of Mass Spectral Data, 8th edition; the NIST Mass Spectral Library (December 2005); and other data sources were utilized to validate the compounds’ identities [30,31].

### 2.2. Quantitative Analysis of Marrubiin

#### 2.2.1. Instrumentation

The standard stock solution and the plant extract under investigation were applied using a CAMAG (Muttenz, Switzerland) Linomat IV sample applicator. In a twin-trough chamber, the plates were saturated with slit dimensions of 6 mm and 0.1 mm (length and width, respectively). The scanning rate was adjusted to 10 mm/s, and the monochromator band width was 20 nm. The band quantification was completed using a densitometer attached to a CAMAG TLC Scanner III. It was operated using a deuterium source and CATS version 4 X software in the absorption mode.

#### 2.2.2. Chemicals

Standard marrubiin was provided by European Pharmacopoeia Reference Standard. All solvents used for extraction and development (chloroform, methanol, acetone, and benzene) were purchased from Merck in Darmstadt, Germany. The 0.25 mm pre-coated silica gel F254 was purchased from the same company. Anisaldehyde/conc. H_2_SO_4_ was used as a spray reagent (prepared by the addition of 50 mL of glacial acetic acid, 0.5 mL of p-anisaldehyde, and 1 mL of concentrated sulfuric acid and kept in the fridge).

#### 2.2.3. Preparation of Plant Extract

The dried extract was precisely weighed out at 100 mg and dissolved in 8 mL of methanol. Methanol was used to adjust the volume before being placed in a 10 mL volumetric flask and kept in the fridge until required.

#### 2.2.4. Standard Solution

**Figure 1:**
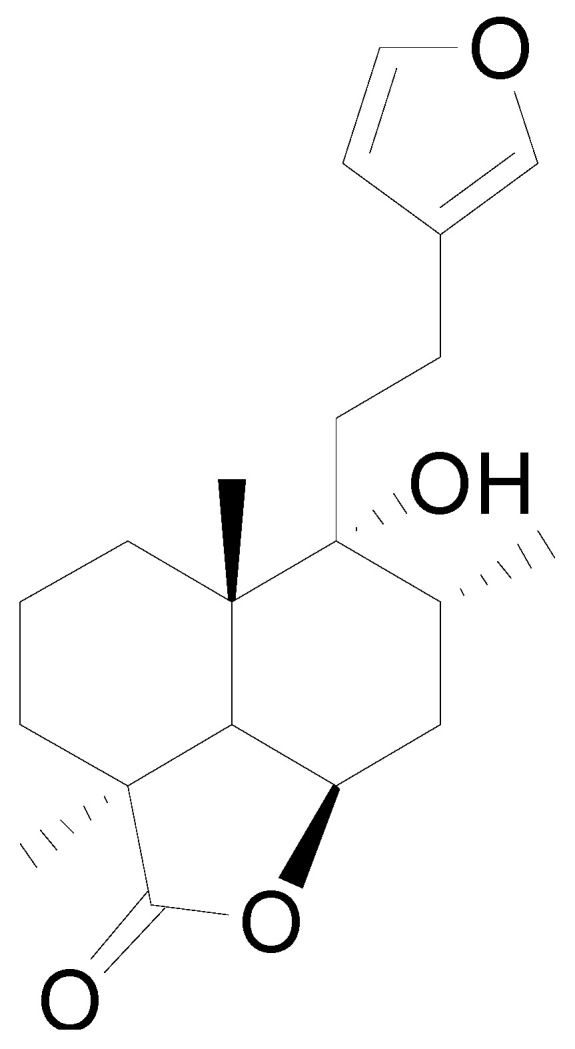
Marrubiin’s chemical structure.

Ten milligrams of the authentic l marrubiin (Figure 1) were weighed, dissolved in eight milliliters of methanol, and then transferred to a ten milliliter volumetric flask to generate a stock solution (A). One mL of stock solution (A) was accurately withdrawn, it was transferred to a 10 mL volumetric flask, and then the volume was adjusted to obtain a stock solution (B). Both stock solutions (A and B) were used to create the calibration curve and kept in the fridge until needed.

#### 2.2.5. Calibration Graph

The calibration curve was set based on the instructions in the ICH guidelines [32]. The dimensions of the used plate were 20 cm × 10 cm. Stock solutions A and B were used to apply different concentrations of marrubiin in 6 mm-long bands. The distance interval between the successive bands was 4 mm. The distance from the plate’s bottom and side edges was 10 mm. At 15 μL/s, the bands were applied. A variety of solvent solutions was used in order to generate the bands, including chloroform:methanol (9:1 and 9.5:0.5) and petroleum ether:ethyl acetate (8:2) and benzene: acetone (9.5:0.5). The solvent mixture which produced uniform tight bands with a proper R_F_ (0.6 ± 0.02) is benzene:acetone (9.5:0.5). Saturation of the chamber was carried out for 20 min. For 15 min, development was carried out, and this was followed by drying the plates for 15 min.

Using the CAMAG TLC scanner, the bands were quantified by linear densitometric scanning in the absorbance mode at different λ ranging from 200 to 280 nm. Anisaldehyde/conc. sulfuric acid was used for spraying the plates, followed by 5 min of heating at 120 °C to yield violet-colored bands, and scanning was conducted at λ 510 nm. Different areas under the peaks with their corresponding concentrations were used to construct the calibration graphs.

#### 2.2.6. Sample Assay

After preparing *M. alysson* L. extract according to the previously mentioned instructions, aliquots of various extract concentrations and the marrubiin standard solution were applied (three times) and developed according to the previous instructions. Anisaldehyde/conc. sulfuric acid was sprayed over the developed zones of the plates after development and drying, and these developed zones were scanned at the indicated wavelengths both before and after spraying (Figure S1a,b).

### 2.3. In Vitro Biological Study: Acetylcholinesterase Inhibitory Effect

#### 2.3.1. Donepezil Standard

Standard donepezil was prepared in the following final concentrations: 0.0001, 0.001, 0.01, 0.1, and 1 µM.

#### 2.3.2. Sample Preparation

Samples of crude *M. alysson* L. extract and the non-polar fraction were prepared in the listed final concentrations: 200, 100, 50, 10, and 5 µg/mL. Marrubiin samples were produced in the listed final concentrations: 200, 100, 50, 10, and 5 µM by dissolving the samples initially in DMSO and diluting them to the desired concentrations with methanol. DMSO has no inhibition properties at this concentration on acetylcholinesterase enzyme for acetylcholinesterase (AChE) inhibitory assay.

#### 2.3.3. Sources of Chemicals and Enzyme

The enzyme acetylcholinesterase was purchased from Sigma-Aldrich (St. Louis, MO, USA) from *Electrophorus electricus*, cat number 3389. The substrate acetylthiocholine iodide and the indicator 3,3′-dithiodipropionic acid di (N-hydroxysuccinimide ester) (DTNB) were purchased from Sigma-Aldrich.

#### 2.3.4. Acetylcholinesterase Inhibitor Assay

According to the procedure mentioned by Ellmann, the assay was conducted with slight variations and modifications [33,34]. Briefly, 10 µL of the indicator solution (0.4 mM in buffer (1): 100 mM tris buffer pH = 7.5) were transferred to a 96-well plate followed by 20 µL of enzyme solution (acetylcholine esterase 0.02 U/mL final concentration in buffer (2): 50 mM tris buffer pH = 7.5 containing 0.1% bovine serum albumin). Then, 20 µL of the sample/standard solution were added followed by 140 µL of buffer (1). The mixture was left to stand at room temperature for 15 min. Afterwards, 10 µL of the substrate (0.4 mM acetylcholine iodide buffer (1)) were then added to each well immediately followed by incubation for 20 min at room temperature in a dark chamber environment. Using a FluoStar Omega microplate spectrophotometer for 30 min at 412 nm, the absorbance of the colored final product was determined after the enzymatic reaction started and the incubation period was complete. Each test was carried out three times. The results of three independent trials are expressed as mean ± SD (IC_50_ ± SD (µM)). By subtracting the absorbance of each test sample’s corresponding blank, the absorbance of the test samples was adjusted.

The formula below was used to determine the percentage inhibition:

$$\frac{Absorbance\;of\;control-Absorbance\;of\;sample}{Absorbance\;of\;control}\times 100$$

The increase in yellow color that thiocholine produces when it combines with dithiobisnitrobenzoate ions was used to determine the enzyme activity. The method’s fundamental step is the determination of the rate at which acetylthiocholine is degraded to produce thiocholine according to how the subsequent reactions couple:

Acetylthiochoine→thiocholine+acetate.Followed by: Thiocholine+dithiobisnitrobenzoate→yellow color.

#### 2.3.5. Micro Plate Reader Analysis

FluoStar Omega, a microplate reader, was used to record the results.

### 2.4. Molecular Docking Studies

Investigations into molecular modeling were carried out on Linux-based systems utilizing Chimera-UCSF and AutoDock Vina. The binding sites inside the proteins were established using the grid-box dimensions around the co-crystallized ligands, and the protein and compound structures were built and optimized using Maestro. Using AutoDock Vina software, the tested drug was docked against the AChE protein structures (PDB = 4M0E) according to the method reported by Nafie and Kishk [35,36].

The interpretation of the molecular docking results was performed based on the binding energy and ligand–receptor interactions. The visualization was then carried out using Chimaera.

Ions and water molecules were removed from the protein receptor. Additionally, hydrogens atoms were added to the protein. Optimization of amino acid residues was completed, including for those that had some atoms in other positions or had atoms missing. The chemical structure of the ligand was optimized by first changing the sequence of the bonds, adding charges, and then adding hydrogen. Additionally, conformational search was used to minimize the energy, or at the very least, clean up the geometry. Prior to the addition of hydrogen, bond order and charges were modified.

Visualization of the interactions between the primary ligand and the receptor with regards to the hydrogen bond and lipophilic interactions with different essential amino acid residues was carried out, as well as the arrangement of the primary (co-crystallized) ligand.

The fundamental criteria indicated in “A Medicinal Chemist’s Guide to Molecular Interactions” were used to carefully examine both interactions. The co-crystallized ligand was docked in the appropriate matched protein using different docking calculations. The results for the ligand and target were assessed using the root mean square deviation (RMSD) between the reference location of the co-crystallized ligand and the docked one anticipated by different docking simulations. The Triangle Matcher placement technique default was selected for docking. The GBVI/WSA dG scoring system, which determines the free energy of the binding of the ligand from a given stance, was used to rank the final postures. In the ligand complex, the protein with the lowest S_score was chosen. When the ligand was redocked with the target, the RMSD was discovered to be 0.606, indicating that it adhered to the same pocket and proving the reliability of the docking parameters.

## 3. Results

### 3.1. GC-MS Analysis of n-Hexane Extract

Nine fatty acid methyl esters were identified by GC-MS analysis of the saponifiable portion of the n-hexane fraction of *M. alysson* L. (Table 1). The methyl ester derivatives of the saturated fatty acids accounted for 50% of the total fatty acids in the *M. alysson* L. lipoid moiety, which was equal to the amount of unsaturated fatty acids. According to the obtained GC chromatogram (Figure S2), the main derivative of saturated fatty acid methyl ester (with an area of over 8%) was hexadecanoic acid methyl ester (palmitic acid methyl ester) (19.31%), which was previously reported for its various activities [6,37,38], whereas the main unsaturated fatty acid methyl esters were recorded as 9,12,15-octadecatrienoic acid methyl ester (Z,Z,Z) (linolenic acid methyl ester) (36.99%) and 9,12-octadecadienoic acid (Z,Z) methyl ester (18.75%), which have also been reported due to their various activities [39,40]. Figure S3 shows the mass spectra of these main derivatives of fatty acid methyl esters. In addition, different saturated and unsaturated fatty acids were identified in the saponifiable fraction of *M. alysson* L. (Table 1), including 9-octadecenoic acid methyl ester (E)- (elaidic acid methyl ester) (7.29%), methyl stearate (octadecanoic acid methyl ester) (3.47%), 2-propenoic acid 3-(4-hydroxyphenyl)- methyl ester (1.32%), eicosanoic acid methyl ester (methyl arachisate) (1.31%), Behenic acid, methyl ester (Methyl behenate) (1.31%), and methyl tetradecanoate (tetradecanoic acid methyl ester) (myristic acid methyl ester) (0.69%).

The GC chromatogram of the unsaponifiable matter USM of *M. alysson L.* hexane fraction (Figure S4) demonstrated the presence of 7 compounds (Table 2). The most common compound was phytol (area 10.01%). Anxiolytic, cytotoxic, antioxidant, autophagy and apoptosis-inducing, antinociceptive, anti-inflammatory, immune-modulating, and antibacterial activities are the biological actions exerted by phytol [41]. The mass spectrum of the major compound, phytol constructed by GC-MS was displayed in Figure S5. Other compounds from different classes were recognized in USM of *M. alysson* L. (Table 2) as; Butylated Hydroxytoluene; 2-Pentadecanone, 6,10,14-trimethyl-; n-Hexadecanoic acid; 9,12,15-Octadecatrienoic acid, (Z,Z,Z), (Linolenic acid); 1a,2,5,5-Tetramethyl-cis-1a,4a,5,6,7,8-hexahydro-gamma-chromene and Card-20(22)-enolide, 3,5,14,19-tetrahydroxy-, (3.ß.,5.ß.)

### 3.2. Quantitative Analysis of Marrubiin

#### 3.2.1. Linearity

After analyzing the different applied concentrations of marrubiin, the wavelength of 254 nm showed a linearity range equal to 100–350 ng/band. Upon investigation of the results obtained after spraying with anisaldehyde/conc. sulfuric acid and heating the same plates, the linearity range was found to be 30–500 ng/band, exhibiting more sensitivity and a wider range of linearity. Accordingly, it was used for the determination of the concentration of marrubiin in the plant extract and the method was validated. The linear regression equation was y = 203.39x + 7476, where y is the spot area and x is the concentration of marrubiin. The correlation coefficient (R^2^) was 0.997.

#### 3.2.2. System Precision

This was evaluated by assessment of a certain concentration (30 ng/band) of the authentic marrubiin carried out six times. The percent relative standard deviation (%RSD) was estimated to be 2.82, indicating the precision of the method.

#### 3.2.3. Method Precision

This was determined by conducting the previously described technique on the plant extract six times and then repeating the analysis. The %RSD was found to be 3.02.

#### 3.2.4. Accuracy

The protocol accuracy was evaluated by recovery after the addition of 10 and 20 ng/band of marrubiin to a previously analyzed one with a concentration of 100 ng/band. Each application was performed in triplicate and the recovery was calculated to be 96.4 and 97.7%, respectively.

#### 3.2.5. Limit of Detection (LOD) and Limit of Quantification (LOQ)

The limit of detection and the limit of quantification were calculated according to the following equations: 3 σ/S and 10 σ/S for the limits of detection and quantification, respectively. σ stands for the standard deviation of the response and S is the slope of the calibration curve. The values were found to be 10 and 35 ng for the LOD and LOQ, respectively.

#### 3.2.6. Robustness

By carrying out the experiment, the procedure’s robustness was ensured with minor changes in the specified conditions. The solvent mixture composition was changed to benzene: acetone (9.6:0.4 and 9.4:0.6) and the % RSD was found to be 2.41 and 3.14, respectively. The time for drying the plates was changed to 10 and 20 min, respectively, and the % RSD was calculated to be 3.41 and 2.98, respectively.

#### 3.2.7. Analytical Solution Stability

The standard solution of marrubiin was kept at 24 h at room temperature and was stored in a refrigerator at 4 °C. In both cases, when the experiment was repeated, the results were not affected, indicating the stability of the solution under the aforementioned conditions.

#### 3.2.8. Sample Analysis

After application of the proposed method (by derivatization with anisaldehyde/conc. sulfuric acid), the concentration of marrubiin in *M. alysson* L. extract was determined based on the regression equation and was found to be 14.09 mg/g of dry extract.

### 3.3. In Vitro Biological Study: Acetylcholinesterase Inhibitory Effect

Experimental evidence indicates that cholinesterase inhibition has become the promising strategy to treat the symptoms of Alzheimer’s disease. AChE accelerates Aβ peptide aggregation and leads to the formation of an Aβ -AChE complex at the synaptic region of the hippocampus, leading to the occurrence of AD [42]. Crude methanolic extract of *M. alysson* L., as well as the non-polar fraction and the pure compound of marrubiin, were evaluated and screened in vitro for their inhibitory activities against AChE using the acetylcholinesterase inhibitory assay by Ellman and Osman, and the results were recorded using a FluoStar Omega microplate reader [33,34].

**Figure 2:**
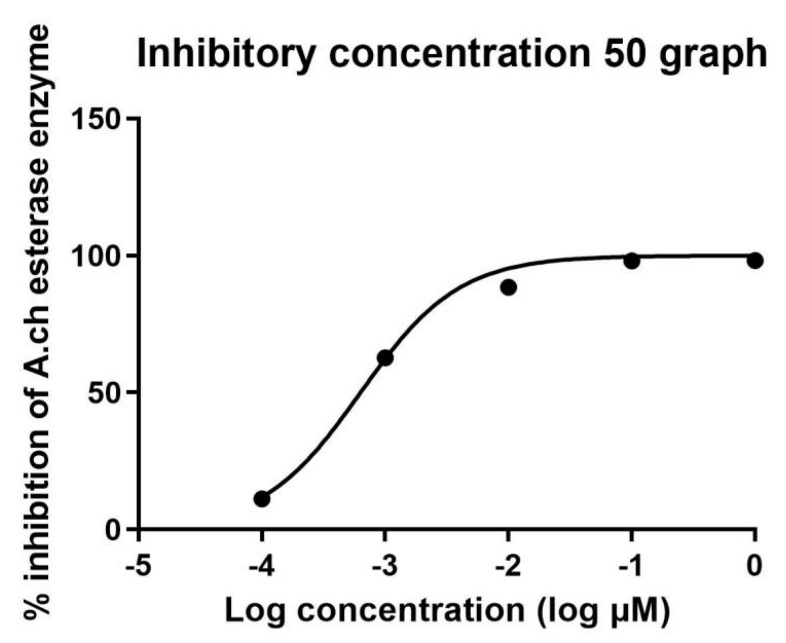
Nonlinear Weaver–Burk plot of cholinesterase inhibition by donepezil.

**Figure 3:**
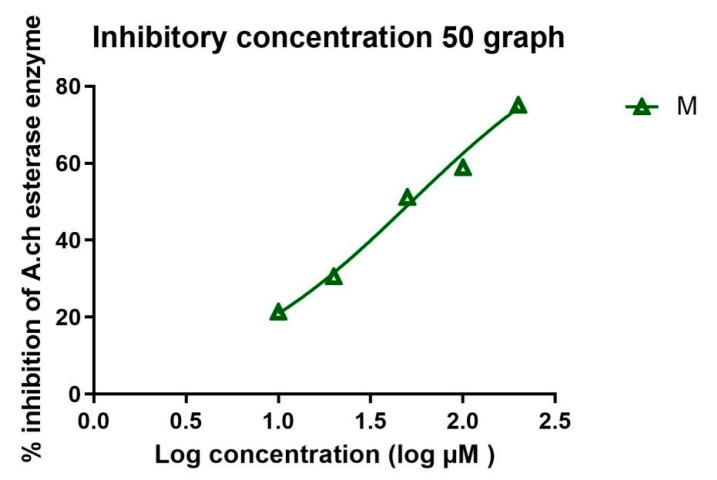
Nonlinear Weaver–Burk plot of cholinesterase inhibition by the pure compound marrubiin.

**Figure 4:**
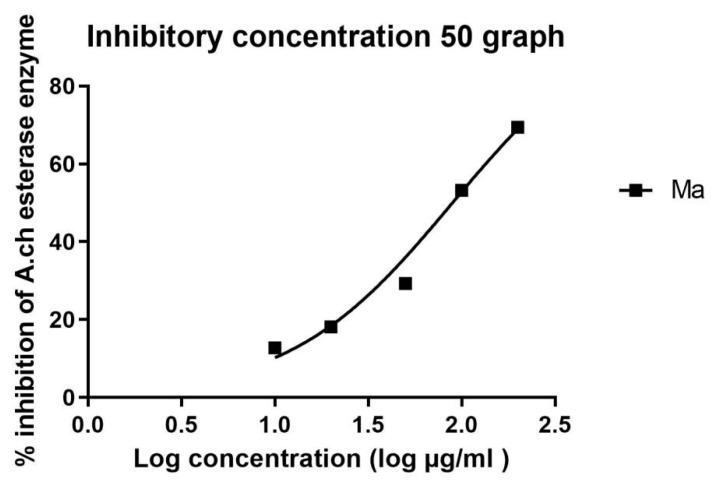
Nonlinear Weaver–Burk plot of cholinesterase inhibition by the crude methanolic *M. alysson* L. extract.

**Figure 5:**
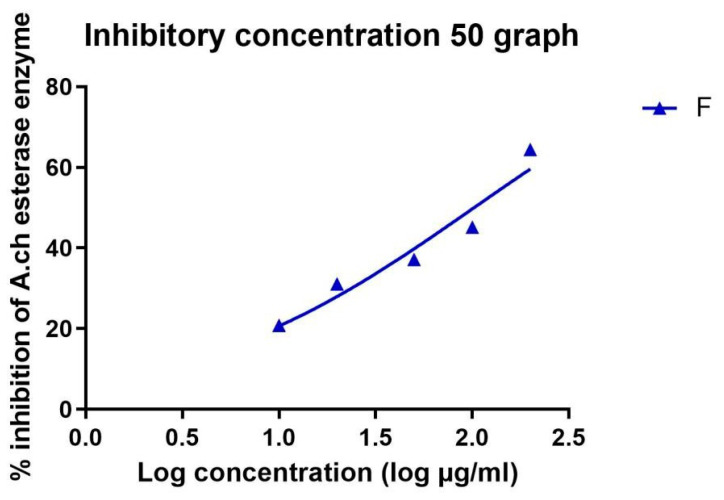
Nonlinear Weaver–Burk plot of cholinesterase inhibition by the non-polar extract.

The IC_50_ in µM was calculated in all extracts and is recorded in Table 3 and Table 4. Standard donepezil displayed IC_50_: 3.387 ± 0.364 nM, and a nonlinear Weaver–Burk plot of cholinesterase inhibition by standard donepezil on AChE was generated, as seen in Figure 2**.** AChE inhibitory action with the highest potency was displayed with the pure compound marrubiin, with an IC_50_ value of 52.66 (µM), as shown in Figure 3. The crude methanolic extract exhibited effective inhibition of the AChE enzyme with an IC_50_ value = 89.31 (µg/mL), as shown in Figure 4. The remaining non-polar fraction also exhibited good AChE inhibitory activity, with an IC_50_ value = 102.2 (µg/mL), as shown in Figure 5.

### 3.4. Molecular Docking

The main fruitfulness of molecular modeling is for the design of promising bioactive leads, as it serves as a useful tool for predicting the interactions of small molecules with drug targets, which can guide synthesis decisions. Natural product-based drug discovery helps to identify the active compounds to be further isolated, characterized, and biologically tested. In this study, all identified compounds in *M. alysson* L. were screened for their binding affinity towards the ACHE protein.

**Figure 6:**
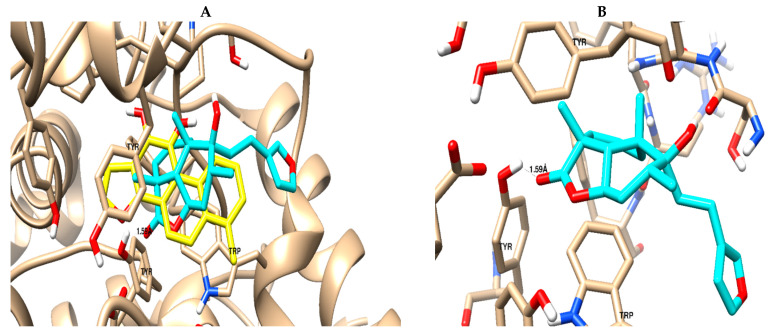
Binding mode and ligand–receptor interactions of the co-crystallized ligand: yellow-colored (**A**) and marrubiin; cyan-colored (**B**) towards the binding site of the acetyl-choline esterase protein.

Marrubiin was screened for binding activity towards the AChE binding site using molecular docking. As summarized in Table 5, marrubiin exhibited good binding affinity, with a binding energy of −19.3 Kcal/mol, and formed a good binding interaction (1 H-bond with Tyr 124), in addition to the Van der Waals forces like the co-crystallized ligand, as seen in Figure 6. This finding was confirmed by the in vitro assay, as previously stated.

## 4. Discussion

The phytochemical study of the non-polar fraction of *M*. *alysson* that was investigated using GC-MS analysis led to the identification of nine fatty acid methyl esters in the saponifiable portion. The main derivatives of fatty acid methyl esters were Hexadecanoic acid, methyl ester (Palmitic acid, methyl ester) (19.31%), 9,12-Octadecadienoic acid (Z,Z)-, methyl ester (Linoleic acid methyl ester) (18.75%) and 9,12,15-Octadecatrienoic acid, methyl ester, (Z,Z,Z)- (Linolenic acid, methyl ester) (36.99%). As mentioned previously in the literature, these three fatty acid methyl esters were the major constituents found in *Marrubium parviflorum*, another species in the genous *Marrubium* when it was investigated by GC-MS with the percentage of 45.8%, 11.26% and 7.28% respectively [43].

Widely in other genus in the family Lamiaceae, GC/MS analysis of the saponifiable matter of *Moluccella laevis* L. aerial parts revealed the presence of linolenic acid, methyl ester (25.58%) and linoleic acid, methyl ester (15.87%) that represented the major proportion of the unsaturated fatty acids. Moreover, Palmitic acid was identified as the major saturated fatty acid with the percentage of 25.04% [44]. Linolenic acid, methyl ester is an essential fatty acid (Omega-3) and had many activities as neuroprotective, anti-inflammatory, and stroke risk reduction. Furthermore, it has antiarrhythmic effects and prevents motor neuron necrosis and apoptosis in spinal injuries by inducing protection against ischemia [45]. Likewise, palmitic acid methyl ester had several biological activities as analgesic and anti-inflammatory. Moreover, it exhibited no cytotoxicity to normal HDF cells but specific cytotoxicity to human leukemic cells. Also, it demonstrated in vivo anticancer efficacy in mice and caused apoptosis in the human leukemic cell line MOLT-4 [46].

On the other hand, the GC chromatogram of the unsaponifiable matter USM of *M*. *alysson* revealed the presence of seven compounds. Phytol was the most predominant compound of the USM portion with percentage of 10.01%. Phytol was previously found in *Marrubium globosum* with the ratio of 2.9% and in *Marrubium cuneatum* with a ratio of 3.2% [47]. In 2021, Aebisher et al reported the presence of phytol (0.4%) in *Mentha piperita,* family Lamiaceae [48]. In 2019, phytol also was reported in *Garden thyme,* family Lamiaceae [49]. In medicinal fields, phytol has shown antioxidant and antinociceptive activities [50] together with anti-allergic and anti-inflammatory properties [51]. Earlier studies also, have revealed that phytol is an excellent immunostimulant [52]. Besides, Phytol has shown antimicrobial activity against *Staphylococcus aureus* and *Mycobacterium tuberculosis*. In addition earlier results suggest that phytol has antischistosomal activities [53].

Notably, marrubiin, which is one of the most prominent metabolites of *Marrubium alysson* L., was not detected through our adopted GC/MS technique. Thus, further characterization was proceeded through simple and rapid HPTLC analysis which successfully provided a sensitive and intelligible chromatographic approach for the analysis of marrubiin within the plant extract. After validation of the method in accordance with ICH criteria guidelines, the results revealed that the used method was highly accurate and precise. Furthermore, robustness and limits of detection and quantification were found to be satisfactory. Using anisaldehyde-conc. sulfuric acid to visualize the spots successfully increased the method’s sensitivity, then scanning at λ = 510 nm. The concentration of marrubiin in *M. alysson* L. extract was found to be 14.09 mg/g of dry extract based on the regression equation.

The crude methanolic extract of *M. alysson* L., non-polar fraction and marrubiin were tested for their acetylcholinesterase (AChE) inhibitory activity. Both methanolic extract of the crude *M. alysson* L. extract and the non-polar fraction displayed reasonable inhibitory activity against AChE. Moreover, results showed marrubiin’s relevant inhibition activity on the same enzyme with IC_50_ value of 52.66 (µM). Despite the modest inhibition activity of marrubiin on AChE, the compound can be considered of clinical significance owing to several reasons. Firstly, one of the prototypes of AChE inhibitor, pyridostigmine, has been reported with moderate pharmacological activity (AChE reversible inhibition activity IC_50_ = 40 μM; being of close IC_50_ to marrubiin), yet being marketed and FDA-approved for management of myasthenia gravis owing to its clinical efficient in controlling the reversing muscle weakness while possessing safe profile (**Cleveland Clinic**; https://my.clevelandclinic.org/health/drugs/18042-pyridostigmine-tablets-regular-release).

On the other hand, more potent AChE inhibitor drugs, such as tacrine, was market withdrawn owing to hepatotoxicity adverse effects despite being strong pharmacologically active down to nanomolar concentrations (AChE IC_50_ = 0.5 μM). In this regards, marrubiin can harbor potential clinical advent for managing medical conditions as long as being tolerable by patients. Marrubiin has been reported with high safety margins [9] with limits up to 100 mg/kg body weight when injected into mice [12]. Additionally, the compound has been reported without any significant cytotoxic activities against 66 examined cancer cell lines as per the **NIH PubMed website** (http://pubchem.ncbi.nlm.nih.gov/summary/summary.cgi?cid=73401).

Besides its reported safety profile, the compound is adherent to Lipinski’s rule of five the thing that prospect its clinical success through the rigors of clinical trials as it possesses similar physiochemical properties to those of 95% of oral market drugs [54]. Marrubiin with high GI absorption (Abbott bioavailability score = 0.55 SWISSADME platform), where this is absolutely beneficial since tacrine withdrawal highlighted owing to its poor oral bioavailability necessitating four-times daily dosing shows prospection for potential drug development and modifications. It projects structural modifications owing to its free OH group serving as versatile moiety for introducing different functionalities. At this point, investigating the marrubiin-target interaction down to its molecular levels through docking analysis would be a suitable tool for future modifications and optimization. Based on predicted residue-wise binding interactions and pocket accommodation, structure-based drug design guidance would be accomplished. Thus, within our molecular docking study, we aimed to highlight the virtual mechanism of binding towards AChE protein to pinpoint the key important regions for the activity are, to be able to predict new derivatives with enhanced activity within future studies. Marrubiin exhibited good binding affinity with binding energy of -19.3 Kcal/mol and formed good binding interaction (1H-bond with Tyr 124). These results may possibly suggest a mechanistic course of action for its acetylcholine esterase inhibitory activity. In these regards, marrubiin was shown to possess potential value as a convenient supply of antiacetylcholinesterase agents in pharmaceutical or nutraceutical compositions with positive health effects. Enhancing cholinergic function through the use of cholinesterase inhibitors is one of the most promising therapeutic approaches for Alzheimer’s disease.

## 5. Conclusions

In conclusion, marrubiin in *M. alysson* plant extract was analyzed using the HPTLC method. In addition, the phytochemical composition of the non-polar fraction of *M. alysson* was investigated using GC-MS analysis. The crude methanolic extract of *M. alysson* L., non-polar fraction, and marrubiin were tested for their acetylcholinesterase inhibitory activity. The study highlighted the potential role of marrubiin as promising lead compound for future investigation and lead optimization adding to the knowledge quest for managing human medical conditions including Alzheimer’s disease.

## Figures and Tables

**Table 1:** *Marrubium alysson* L. fatty acid methyl esters.

	Retention Time (min)	Retention Index		Name and Structure of Compound	Molecular Formula	Molecular Weight
		(Cal.)	(Rep.)*			
1	28.960	1688	1688	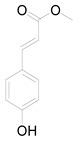 2-Propenoic acid, 3-(4-hydroxyphenyl)-, methyl ester	C_10_H_10_O_3_	178.1
2	29.275	1726	1727	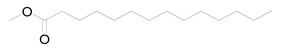 Methyl tetradecanoate (Tetradecanoic acid, methyl ester) (Myristic acid, methyl ester)	C_15_H_30_O_2_	242.3
3	33.625	1926	1928	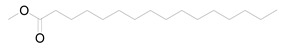 Hexadecanoic acid, methyl ester (Palmitic acid, methyl ester)	C_17_H_34_O_2_	270.4
4	36.840	2089	2093	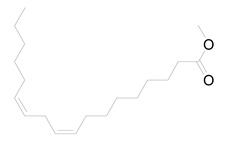 9,12-Octadecadienoic acid (Z,Z)-, methyl ester (Linoleic acid, methyl ester)	C_19_H_34_O_2_	294.4
5	36.920	2098	2100	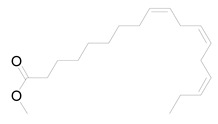 9,12,15-Octadecatrienoic acid, methyl ester, (Z,Z,Z)- (Linolenic acid, methyl ester)	C_19_H_32_O_2_	292.4
6	37.005	2110	2110	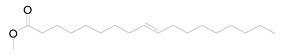 9-Octadecenoic acid, methyl ester, (E)- (Elaidic acid, methyl ester)	C_19_H_36_O_2_	296.4
7	37.560	2126	2128	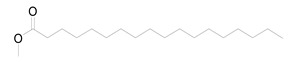 Methyl stearate (Octadecanoic acid, methyl ester)	C_19_H_38_O_2_	298.5
8	41.175	2324	2324	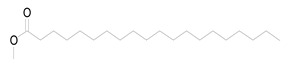 Eicosanoic acid, methyl ester (Methyl arachisate)	C_21_H_42_O_2_	326.5
9	44.505	2524	2527	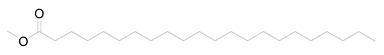 Behenic acid, methyl ester (Methyl behenate)	C_23_H_46_O_2_	354.6

* Compounds are listed based on their calculated retention indices (Cal.) experimentally on the RTX-5 column relative to standard mixtures of hydrocarbons (C_7_-C_40_). The compounds identification were based on comparison of their reported retention indices (Rep.) and mass spectral data.

**Table 2:** Chemical composition of the unsaponifiable matter (USM) of *M. alysson* L.

	Retention Time (min)	Retention Index		Name and Structure of Compound	Molecular Formula	Molecular Weight
		(Cal.)	(Rep.)*			
1	24.045	1512	1511	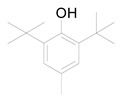 Butylated Hydroxytoluene	C_15_H_24_O	220.3
2	31.940	1846	1845	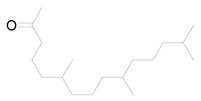 2-Pentadecanone, 6,10,14-trimethyl-	C_18_H_36_O	268.4
3	34.260	1984	1978	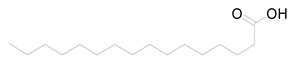 n-Hexadecanoic acid	C_16_H_32_O_2_	256.4
4	37.350	2148	2145	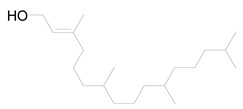 Phytol	C_20_H_40_O	296.5
5	37.535	2162	2159	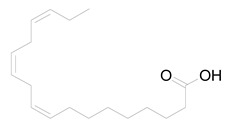 9,12,15-Octadecatrienoic acid, (Z,Z,Z), (Linolenic acid)	C_18_H_30_O_2_	278.4
6	39.230	2231	nd	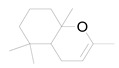 1a,2,5,5-Tetramethyl-cis-1a,4a,5,6,7,8-hexahydro-gamma-chromene	C_13_H_22_O	194.3
7	54.300	3238	nd	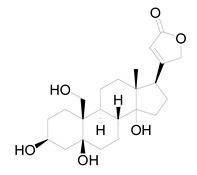 Card-20(22)-enolide, 3,5,14,19-tetrahydroxy-, (3.ß.,5.ß.)	C_23_H_34_O_6_	406.5

* Compounds are listed based on their calculated retention indices (Cal.) experimentally on the RTX-5 column relative to standard mixtures of hydrocarbons (C_7_-C_40_). The compounds identification were based on comparison of their reported retention indices (Rep.) and mass spectral data. nd = No data.

**Table 3:** IC_50_ inhibitory values of the crude methanolic extract, non-polar fraction, and marrubiin.

Sample ID	IC_50_	SD
Crude methanolic extract	89.31 (µg/mL)	7.27
Non-polar fraction	102.2 (µg/mL)	7.60
Marrubiin	52.66 (µM)	5.66

**Table 4:** Summarized IC_50_ data of crude methanolic extract, non-polar fraction, and marrubiin using different final concentrations.

Crude Methanolic Extract					
Conc (µg/mL)	10	20	50	100	200
% Inhibition	13.23683	18.94838	28.40949	49.33729	71.74747
	12.41716	16.5068	33.46704	52.82525	64.07394
	12.62644	19.12278	26.05511	57.53401	72.61946
Av. Inhibition	12.76014	18.19265	29.31055	53.23218	69.48029
**Marrubiin**					
% Inhibition	20.29795	37.5789	51.6946	56.98324	77.43017
	22.97952	25.81006	47.89572	60.18622	74.97207
	20.74488	27.07635	53.85475	61.97393	78.24953
Av. Inhibition	21.34078	30.1551	51.14836	59.71446	76.88392
**Non-Polar Fraction**					
% Inhibition	21.25916	33.03104	37.34253	47.5061	62.63516
	18.38158	28.23509	34.77503	42.88455	64.29194
	22.91594	31.98465	39.57098	45.13463	64.98954
Av. Inhibition	20.85223	31.08359	37.173	45.17509	64.4489

**Table 5:** A summary of the interactions between ligands and receptors that have affinity for the AChE protein.

Name	Binding Energy (Kcal/mol)	Ligand–Receptor Interactions
**Co-crystallized ligand**	−18.6	1 H-bond with Tyr 124 Van der Waals forces with Trp 286 and Tyr 341
**Marrubiin**	−19.34	1 H-bond with Tyr 124 Van der Waals forces with Tyr 341 and Trp 286

## Data Availability

Data are available within the article and the Supplementary Materials.

## Supplementary Materials

The following supporting information can be downloaded at: https://www.mdpi.com/article/10.3390/metabo14010027/s1, Figure S1: Chromatograms of HPTLC scanned at λ = 510 nm (a: standard marrubiin, b: *M*. *alysson* L. extract); Figure S2: Chromatogram of GC-MS analysis of fatty acid methyl esters of *M*. *alysson* L.; Figure S3. Mass spectra of the major phytochemicals listed in Table 1; Figure S4: Chromatogram of GC-MS analysis of unsaponifiable matter of *M*. *alysson* L.; Figure S5: Mass spectrum of phytol, a major phytochemical listed in Table 2.
