# Supplementary material for: Analysis of Marrubiin in Marrubium alysson L. Extract Using Advanced HPTLC: Chemical Profiling, Acetylcholinesterase Inhibitory Activity, and Molecular Docking
Source: Metabolites. 2023 Dec 30;14(1):27. doi: 10.3390/metabo14010027 (PMC10821196; doi:10.3390/metabo14010027)
Supplement: Supplementary file 1 [file metabolites-14-00027-s001.zip › metabolites-2630688-supplementary.pdf]

metabolites

MDPI

Article

Analysis of Marrubiin in *Marrubium alysson* L. Extract Using Advanced HPTLC: Chemical Profiling, Acetylcholinesterase Inhibitory Activity and Molecular Docking

Supplementary Materials

1) **Figure S1.** Chromatograms of HPTLC scanned at λ = 510 nm (a: standard marrubiin, b: *M. alysson* L. extract)
2) **Figure S2.** Chromatogram of GC-MS analysis of fatty acid methyl esters of *M. alysson* L.
3) **Figure S3.** Mass spectra of major phytochemicals listed in Table 1.
4) **Figure S4.** Chromatogram of GC-MS analysis of unsaponifiable matter of *M. alysson* L.
5) **Figure S5.** Mass spectrum of phytol, a major phytochemical listed in Table 2.

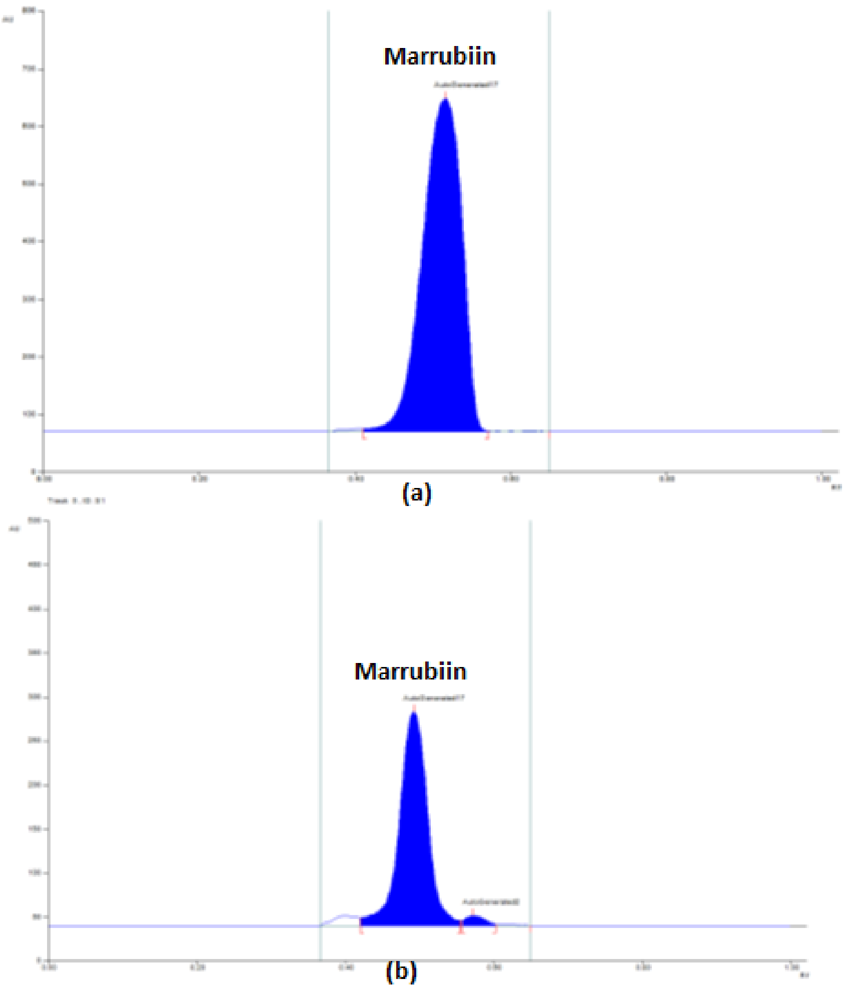

Figure S1. Chromatograms of HPTLC scanned at λ = 510 nm (a: standard marrubiin, b: *M. alysson* L. extract)

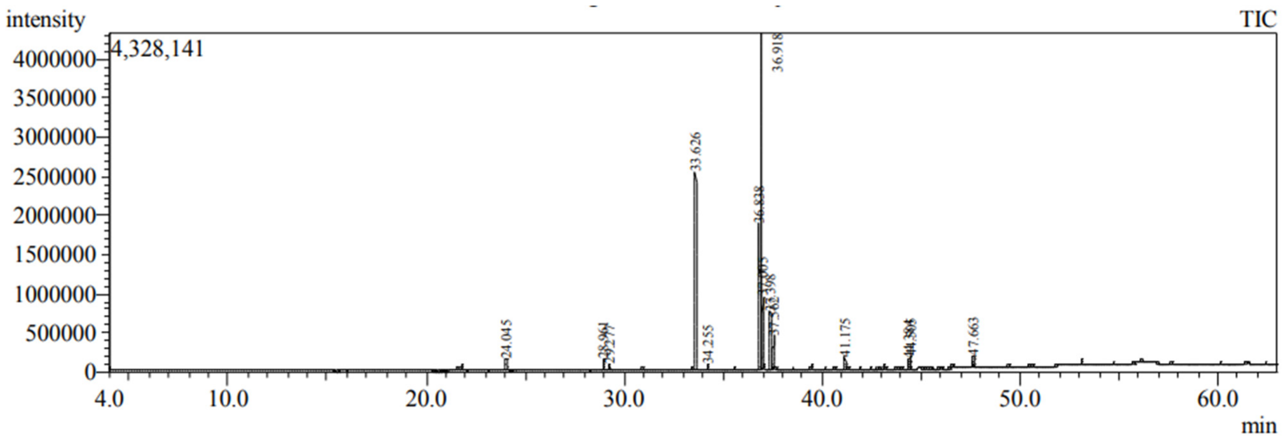

Figure S2. Chromatogram of GC-MS analysis of fatty acid methyl esters of *M. alysson* L.

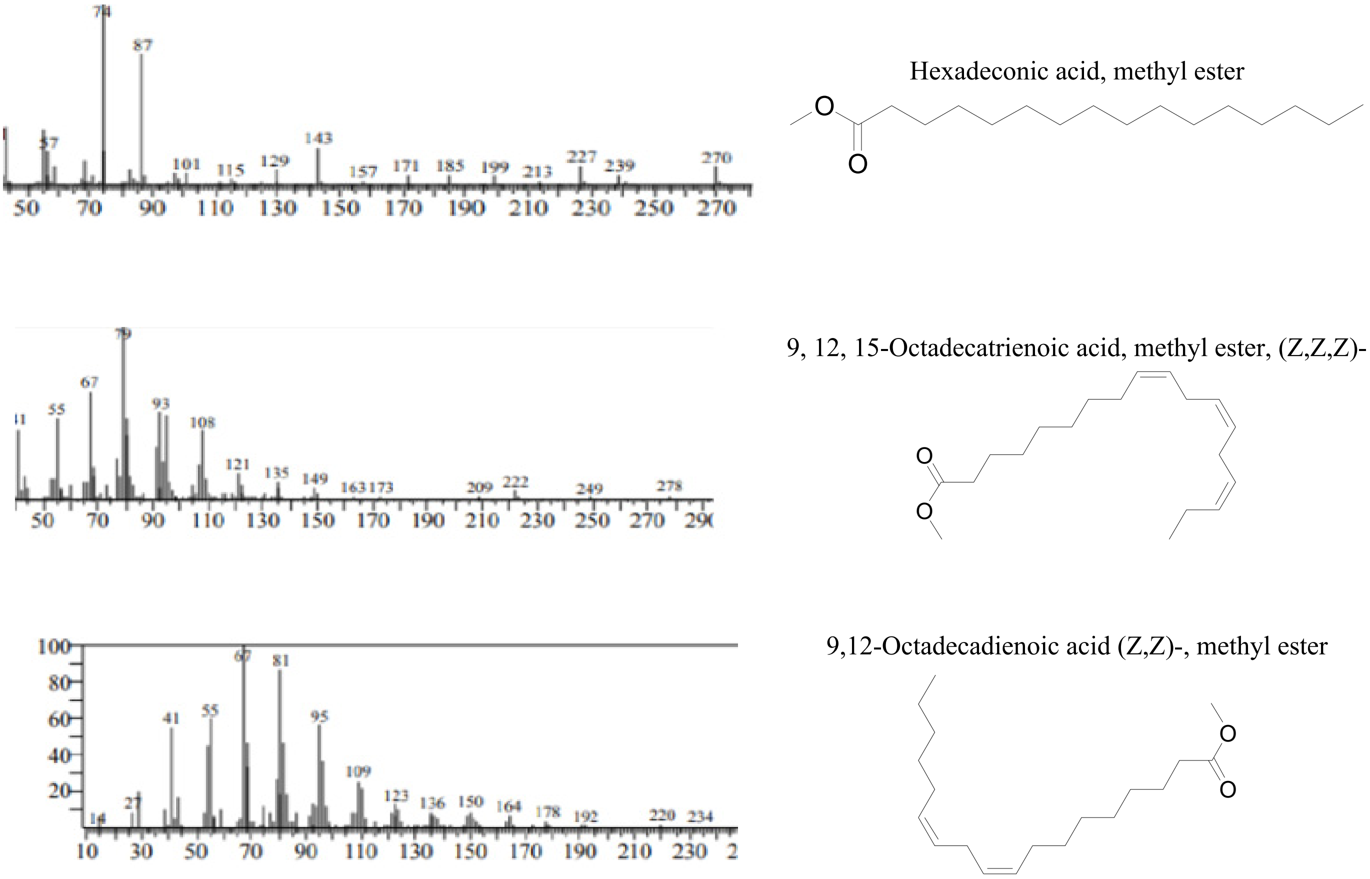

Figure S3. Mass spectra of major phytochemicals listed in Table 1.

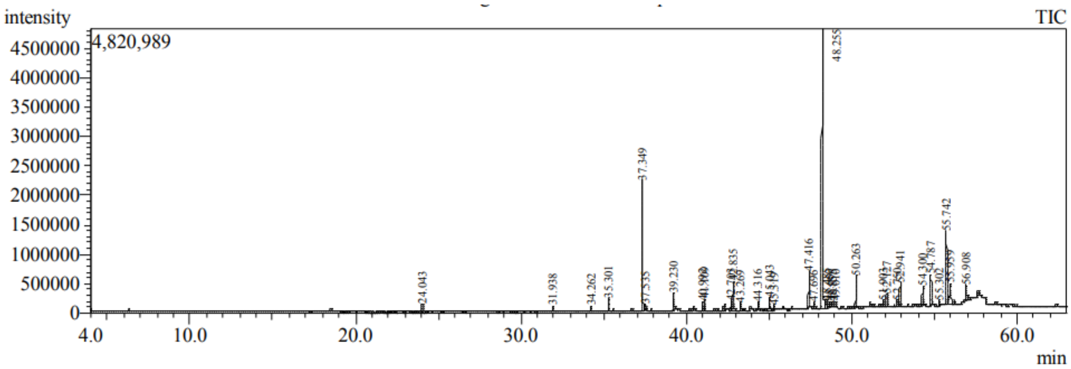

Figure S4. Chromatogram of GC-MS analysis of unsaponifiable matter of *M. alysson L.*

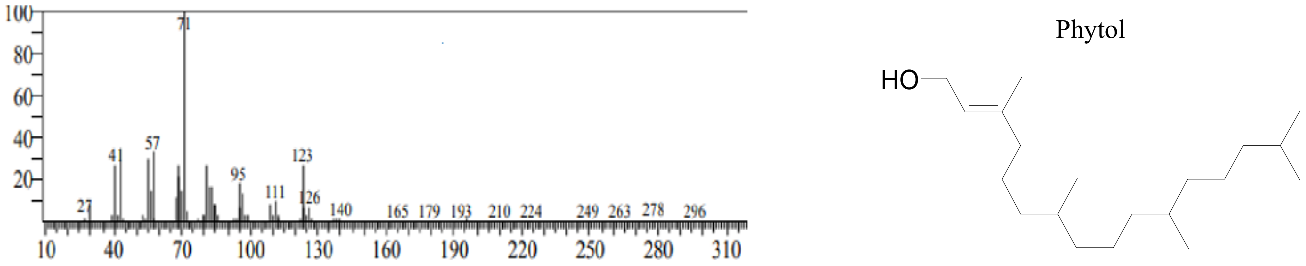

Figure S5. Mass spectrum of phytol, a major phytochemical listed in Table 2.
